# Machine Learning-Based Radiomics Nomogram Using Magnetic Resonance Images for Prediction of Neoadjuvant Chemotherapy Efficacy in Breast Cancer Patients

**DOI:** 10.3389/fonc.2020.01410

**Published:** 2020-08-13

**Authors:** Shujun Chen, Zhenyu Shu, Yongfeng Li, Bo Chen, Lirong Tang, Wenju Mo, Guoliang Shao, Feng Shao

**Affiliations:** ^1^Cancer Hospital of the University of Chinese Academy of Sciences (Zhejiang Cancer Hospital), Hangzhou, China; ^2^Institute of Cancer and Basic Medicine (IBMC), Chinese Academy of Sciences, Hangzhou, China; ^3^Department of Radiology, Zhejiang Cancer Hospital, Hangzhou, China; ^4^Department of Radiology, Zhejiang Provincial People's Hospital, Affiliated People's Hospital of Hangzhou Medical College, Hangzhou, China; ^5^Department of Breast Surgery, Zhejiang Cancer Hospital, Hangzhou, China; ^6^Department of Pathology, Zhejiang Cancer Hospital, Hangzhou, China; ^7^Department of Gynecological Oncology, Zhejiang Cancer Hospital, Hangzhou, China

**Keywords:** radiomics, nomogram, breast cancer, neoadjuvant chemotherapy, pathological complete response, machine learning

## Abstract

**Purpose:** The construction and validation of a radiomics nomogram based on machine learning using magnetic resonance image (MRI) for predicting the efficacy of neoadjuvant chemotherapy (NACT) in patients with breast cancer (BCa).

**Methods:** This retrospective investigation consisted of 158 patients who were diagnosed with BCa and underwent MRI before NACT, of which 33 patients experienced pathological complete response (pCR) by the postoperative pathological examination. The patients with BCa were divided into the training set (*n* = 110) and test set (*n* = 48) randomly. The features were selected by the maximum relevance minimum redundancy (mRMR) and absolute shrinkage and selection operator (LASSO) algorithm in the training set. In return, the radiomics signature was established using machine learning. The predictive score of each patient was calculated using the radiomics signature formula. Finally, the predictive scores and clinical factors were used to perform the multivariate logistic regression and construct the nomogram. Receiver operating characteristics (ROC) analyses were used to assess and validate the diagnostic accuracy of the nomogram in the test set. Lastly, the usefulness of the nomogram was confirmed via decision curve analysis (DCA).

**Results:** The radiomics signature was well-discriminated in the training set [AUC 0.835, specificity 71.32%, and sensitivity 82.61%], and test set (AUC 0.834, specificity 73.21%, and sensitivity 80%). Containing the radiomics signature and hormone status, the radiomics nomogram showed good calibration and discrimination in the training set [AUC 0.888, specificity 79.31%, and sensitivity 86.96%] and test set (AUC 0.879, specificity 82.19%, and sensitivity 83.57%). The decision curve indicated the clinical usefulness of our nomogram.

**Conclusion:** Our radiomics nomogram showed good discrimination in patients with BCa who experience pCR after NACT. The model may aid physicians in predicting how specific patients may respond to BCa treatments in the future.

## Introduction

Currently, breast cancer (BCa) is the most commonly diagnosed malignancy in females worldwide as of 2018, accounting for ~25% of all new diagnoses and nearly 15% of cancer-associated deaths in females (1). This translates to 2.1 million new cases of BCa in 2018, along with more than 600,000 BCa-related deaths. Globally, women have a 5% cumulative risk of being diagnosed with BCa by the age of 75, although the risk varies substantially by country.

Neoadjuvant chemotherapy (NACT) is a central component of BCa therapy (2). In previous studies, NACT has been associated with lower disease stages, increased sensitivity of chemotherapy drugs, improved resection and breast preservation rates, and increased pathological complete response (pCR) in some patients with BCa (3, 4). The disease-free survival (DFS) and overall survival (OS) of BCa patients with pCR are significantly longer than those of patients without pCR (5), suggesting that pCR may be a potential prognostic factor and target of NACT. However, even with the latest advances in chemotherapy regimens, the number of patients with pCR remains low at 12–28% worldwide (6). In non-pCR patients, NACT may fail to produce a full therapeutic effect, which can delay surgical intervention (7). Accordingly, the rapid and effective screening of patients is critically important to identify patients more likely to respond to NACT, which may lead to improved patient outcomes.

In recent years, there has been extensive cross integration of radiation medicine with bioengineering, which has produced the field of radiomics (8, 9). Radiomics is a method for the extraction of high-dimensional data from radiographic medical images using data-characterization algorithms. Recent studies have shown that radiomics features of magnetic resonance imaging (MRI) may allow for the prediction of NACT and radiotherapy response in patients with rectal cancer (10). In addition, the radiomic features from MRI have been used to predict how patients with BCa would respond to NACT before the treatment was initiated (11). Recently, a new multicenter study was conducted to assess a multiparameter prediction model using MRI data that could accurately predict which patients would have pCR before undergoing treatment (12). While radiomics may be used to predict the efficacy and potential benefits of NACT, no studies have evaluated the potential impact of different machine learning techniques on radiomics.

Machine learning involves the building of data-derived computational models and methods to improve the accuracy, performance, or predictive abilities of the model, which is an important part of radiomics (13, 14). Accordingly, machine learning strategies have high prognostic and predictive power, along with excellent stability, all of which are desired for radiomics-based analyses. In this study, our aim was to use a highly predictive and stable machine learning strategy for constructing a radiomics nomogram that could be used to predict pCR in patients with BCa. The radiomics nomogram provides a noninvasive, convenient, and low-cost strategy that may improve the treatment of BCa in the future.

## Materials and Methods

### Patient Information

**Figure 1:**
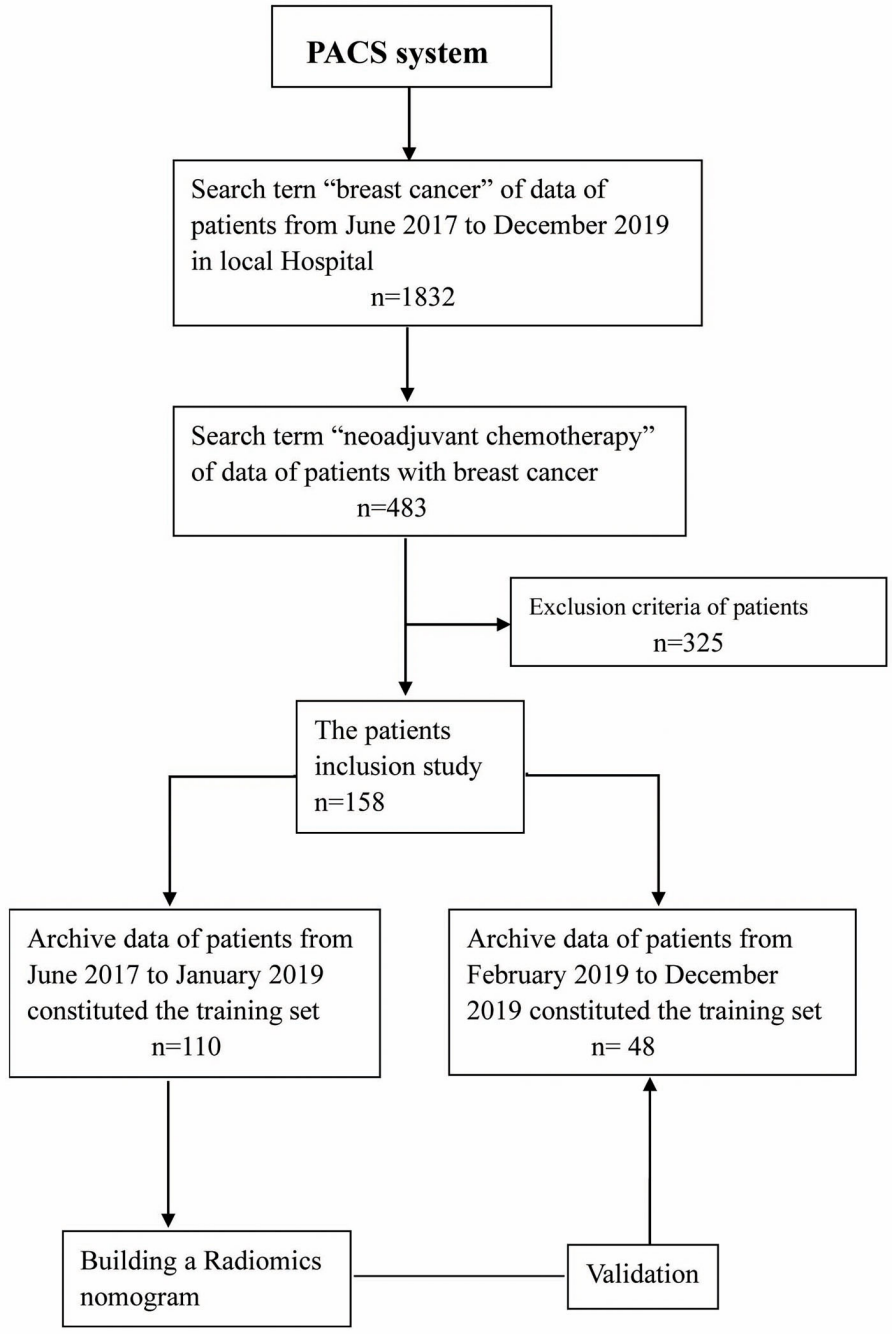
Flowchart showing the recruitment of patients and the overall design of this retrospective study.

This study was approved by the Ethics Committee of Zhejiang Cancer Hospital (Gongshu, P.R. China). The requirement for informed consent was waived due to the retrospective nature of this study. The patients were enrolled in our hospital from June 2017 to December 2019. All the patients underwent MRI within 1–2 weeks prior to NACT. The inclusion criteria for this study were as follows: (a) invasive BCa confirmed by biopsy without distant metastasis; (b) complete NACT was initiated with no prior history of treatment; and (c) surgery was performed after NACT, and pathological evaluation was performed after the operation. The exclusion criteria for this study were as follows: (a) NACT was not completed; (b) surgery was not performed, or postoperative pathology was not evaluated; and (c) the MRI data were unavailable. In addition, the study population was group by 7:3 according to the diagnosis time. The patients from June 2017 to January 2019 were part of the training set (*n* = 110). During the training period, the robustness of the radiomic features was tested, and the model was constructed. The patients from February 2019 to December 2019 were part of the test set (*n* = 48) to verify the reliability of the constructed model. The recruitment path of the subjects and research design of this study are shown in Figure 1.

### MRI Scanning Process and Immunohistochemical Evaluation

All breast MRI scans were performed at a local hospital using the 3.0 Tesla MRI scanner (MAGNETOM Verio A Tim System; Siemens Healthcare, Erlangen, Germany). During the scan, an axial fat-suppressed T2WI sequence and axial diffusion weight imaging (DWI) images were obtained using the two *b*-values of 0 and 1,000 s/mm^2^ before the contrast agent was administered. Initially, a fat-saturated T1WI scan was recorded before injection of the contrast agent or dynamic contrast-enhanced (DCE) scanning were performed. Next, DCE images were acquired as six post-injection scans with intervals of 38 s following the intravenous injection of Magnevist. Next, 0.2 mL/kg of body weight of the gadolinium-based agent (Magnevist; Bayer Healthcare, Berlin, Germany) was injected using an MRI compatible power injector (rate = 2 mL/s), which was flushed with 20-mL of saline using the high-pressure injector. Additional details about the MRI parameters can be found in the Supporting Information.

Immunohistochemistry (IHC) was used to assess the expression of several receptors and antigens commonly associated with BCa from biopsy in the pre NACT, including estrogen receptor (ER), progesterone receptor (PR), human epidermal growth factor receptor 2 (HER2), and antigen Ki67 (Ki-67). Tumors with <1% of nuclear staining were denoted as ER/PR-negative, while ≥1% was denoted as ER/PR-positive. Next, 20% was established as the cutoff value for Ki-67 expression. In terms of HER2 expression, IHC scores of 0 or 1+ were denoted as HER2-negative, and 3+ was denoted as HER2-positive. An IHC score of 2+ required further investigation using *in situ* hybridization (ISH), with non-amplified results being denoted as HER2-negative and amplified results being HER2-positive.

### NACT and the Pathological Response of Treatment

The patients included in this study underwent four or six cycles of NACT before undergoing breast surgery. The National Comprehensive Cancer Network (NCCN) guidelines were used to establish the therapy timeline and procedures (15). In this study, 85% of patients (*n* = 121) were given the taxane-based NACT regimen, while the remaining 15% of patients (*n* = 21) were given the anthracycline and taxane-based NACT regimen. Additionally, the patients found to be HER2-positive were prescribed trastuzumab (loading dose = 8 mg/kg; maintenance dose = 6 mg/kg). All of the patients underwent surgery after NACT, and pathological specimens were evaluated using the Miller-Payne system (16) for the pathological assessment of NACT response. The histopathological examination and analysis were performed by a dedicated breast pathologist, who was blinded to the MRI data, with more than 12 years of experience in the field of breast pathology. Next, the efficacy of NACT was determined by examination and comparison of the specimens from the initial biopsies with those obtained from the radical resection specimens. The Miller-Payne system is divided into five grades. In the current study, grade 5 and the absence of lymph node invasion in the ipsilateral sentinel node or lymph nodes removed during axillary dissection (yPT0/isN0), while the remaining grades were denoted as the non-pCR group. Additional details about the pathological grades can be found in the Supporting Information.

### Segmentation and Preprocessing of MR Images

The DCE-MRI images were imported into the itk-snap software. The third phase, which showed the most apparent enhancement, was selected for sketching tumor boundaries. The largest layer of the tumor was selected for sketching, and areas of necrosis, calcification, and bleeding were avoided. The region of interest (ROI) of the tumor was saved and imported into the DWI images (*b* = 1,000 s/mm^2^) and T2WI images using the replication function. Next, the ROIs of tumors from three different MRI sequences were saved. In addition, manual corrections were further performed to prevent small deviations in delineating the ROI boundary. All tumor sketches were completed by two senior radiologists independently without knowing the pathological results. Radiologist A had ten years of experience, and radiologist B had ~15 years of experience in the study of breast radiology.

The ROIs from the three MRI sequences were loaded into the AK analysis software for feature extraction. The images were initially processed before the feature extraction was performed. The initial processing of images required the resampling of voxels to 1 × 1 × 1 mm^3^ and standardization of gray levels to the 1–256 scale. This eliminated the potential influences of different imaging sequences on the extracted features (17).

### Extraction of Radiomic Features

AK software was used to extract the radiomic features, including the histogram, FormFactor, gray level co-occurrence matrix (GLCM), and run-length matrix (RLM). These features can characterize the heterogeneity of cancer and reflect changes in the tumor microstructure (17). The most robust features were used for manual correction purposes to improve the usefulness of the model (18). The Spearman's rank test was used to assess the correlation coefficients between features of set-A (Radiologist A) and set-B (Radiologist B). Any features that had correlation coefficients > 0.8 were denoted as having “robust” features (19). Three sets of robust features corresponding to T2WI, DWI, and DCE sequences were obtained. The feature values in this study were the average values of feature set-A and feature set-B.

### Establishment of an Optimal Radiomics Signature Based on Machine Learning

The maximum-relevance minimum redundancy (mRMR) algorithm was used to extract the robust features in the training set. Maximum relevance allowed for the selection of features most associated with pCRs (20), while minimum redundancy allowed for the selection of features with minimal redundancy among the others. Optimal features set with high correlation and low redundancy were obtained using the mRMR algorithm. Next, the typical absolute shrinkage and selection operator (LASSO) algorithm allowed for the reduction of dimensions and construction of the radiomics signature through machine learning techniques (21).

The five machine learning classifiers utilized in this study included Support Vector Machine (SVM), Bayes, *k*-Nearest Neighbor (KNN), Random Forest, and Decision Tree. The machine learning models were constructed using five-fold cross-validation. In short, this required that 20% of data be used to test the model, while the other 80% of data were used to create the model. After a total of 10 repeats, the average values were used to estimate the performance of the model. To demonstrate the correlation between the radiomics signature and pCR status, the signature model was used to score the training set in terms of pCR probability. The score was defined as the rad score, and was used to determine the effectiveness of the signature models for differentiating between pCR and non-pCR patients. The formula of the model used in the training set was employed to calculate the scores for the test set. Lastly, the accuracy of the radiomics signature from the training and test sets was evaluated with area under curve (AUC) value of receiver operating characteristic (ROC) curve. In addition, we selected the machine learning method with the largest AUC and the smallest difference between the training and the verification sets as the model construction method of this study. Detailed information about the dimensionality reduction and radiomics signature can be found in the Supporting Information.

### Development and Evaluation of the Radiomics Nomogram

For the training group, univariate logistic regression analyses were performed to select independent predictors of pCR for each potential predictive variable, including clinical factors (i.e., gender, age, and menstrual status), clinical stage of the tumor, biomarker expression (i.e., ER, PR, HER2, and Ki-67), and the rad score. Multivariable logistic regression analyses that combined the independent predictors were applied to develop a pCR prediction model. Next, multivariate logistic regression was used to create the radiomics nomogram.

The variance inflation factor (VIF) was used to diagnose the collinearity of each variable (22) with VIF values >10, indicating severe multicollinearity (23). The calibration performance was evaluated with the calibration curve, and fitness was analyzed by the Hosmer–Lemeshow test. The ROC curve allowed for the estimation of diagnostic accuracy using the nomogram. The probability score for pCR was determined for the patients included in the study using the nomogram, and all patients were divided into high or low probability groups according to the ROC curve cut-off value. The clinical effect of the nomogram was determined using the actual patients with pCR from the different probability groups. The net benefit of the nomogram was determined using the DCA curve (24).

### Statistical Analysis

SPSS 17.0 software (IBM, Chicago, IL, USA) was used to perform the Kolmogorov–Smirnov test for evaluating the normality of the distribution of the data, and the chi-square test for the categorical data. The likelihood ratio test with backward step-down selection was applied to the multivariate logistic regression model. VIFs were calculated using the SPSS 17.0 software. The MedCalc15.8 software (MedCalc, Ostend, Belgium) was used to assess the ROC curves, and differences between various AUCs were compared with the DeLong test. The R statistical software Version 3.4.1 was used for all other statistical analyses. The “mRMRe” and “glmnet” packages were used for mRMR and LASSO analyses. Calibration plots and the radiomics nomogram were established with the “rms” package, and DCA with the “dca.R” package. Two-sided *p* < 0.05 were considered as being statistically significant.

## Results

### Characteristics of Patients in This Study

A flowchart of participant recruitment is presented in Figure 1. No significant differences were detected in the age, menstrual status, clinical stage of the tumor, or biomarker expression (i.e., ER, PR, HER2, and Ki-67) between patients in the training and test sets, as shown in Table 1. However, significant differences in ER expression, PR expression, and the radiomics signature were detected between pCR and non-pCR (all *p* < 0.05). As shown in Table 2, the other differences were insignificant.

**Table 1:** Clinical characteristics of patients in the primary and internal validation cohorts.

**Variables**		**Training set (*****n*** **=** **110)**		**Test set (*****n*** **=** **48)**		***P*-value**
		***n***	**%**	***n***	**%**	
Age (years, mean ± SD)		49.85 ± 8.78		52.96 ± 8.97		0.442
Menstrual status	Premenopausal	68	61.8	38	79.2	0.051
	Postmenopausal	42	38.2	10	20.8	
Histologic type	NST invasive carcinoma	89	80.9	39	81.3	0.96
	Other	21	19.1	9	18.7	
Clinical stage	I	16	14.5	7	14.6	0.954
	II	64	58.2	29	60.4	
	III	30	27.3	12	25	
ER status	Negative	49	44.5	22	45.8	0.881
	Positive	61	55.5	26	54.2	
PR status	Negative	43	39.1	18	37.5	0.85
	Positive	67	60.9	30	62.5	
HER2 status	Negative	51	46.4	15	31.3	0.076
	Positive	59	53.6	33	68.7	
Ki-67	Low	19	17.3	8	16.7	0.926
	High	91	82.7	40	83.3	

*ER, estrogen receptor; PR, progesterone receptor; HER2, human epidermal growth factor receptor 2; Ki-67, antigen Ki67*.

**Table 2:** Clinical characteristics of the training and validation sets of breast cancer (BCa) patients with and without pCR.

**Variable**		**Training set (*****n*** **=** **110)**			**Test set (*****n*** **=** **48)**		
		**pCR (*n* = 23)**	**Non-pCR (*n* = 87)**		**pCR (*n* = 10)**	**Non-pCR (*n* = 38)**	
		***n* (%)**	***n* (%)**	***P*-value**	***n* (%)**	***n* (%)**	***P*-value**
Age (years, mean ± SD)		48.9 ± 10.6	50.1 ± 8.3	0.583	48.8 ± 8.7	54.1 ± 8.8	0.112
Menstrual status	Premenopausal	15 (62.2)	53 (60.9)	0.706	9 (90)	28 (73.7)	0.275
	Postmenopausal	8 (34.8)	34 (39.1)		1 (10)	10 (26.3)	
Histologic type	NST invasive carcinoma	16 (69.6)	73 (83.9)	0.12	8 (80)	31 (81.6)	0.909
	Other	7 (30.4)	14 (16.1)		2 (20)	7 (18.4)	
Clinical stage	I	3 (13)	13 (14.9)	0.812	2 (20)	5 (13.2)	0.993
	II	13 (56.5)	51(58.6)		7 (70)	21 (55.3)	
	III	7 (30.5)	23 (26.5)		1 (10)	12 (31.5)	
ER status	Negative	16 (69.6)	33 (37.9)	0.007^*^	8 (80)	14 (36.8)	0.015^*^
	Positive	7 (30.4)	54 (62.1)		2 (20)	24 (63.2)	
PR status	Negative	16 (69.6)	27 (31)	0.001^*^	6(60)	9 (23.7)	0.027^*^
	Positive	7 (30.4)	60 (69)		4 (40)	29 (76.3)	
HER2 status	Negative	14 (60.9)	37 (42.5)	0.117	6 (60)	12 (31.6)	0.099
	Positive	9 (39.1)	50 (57.5)		4 (40)	26 (68.4)	
Ki-67	Low	5 (21.7)	14 (16.1)	0.524	2 (20)	6 (15.8)	0.751
	High	18 (78.3)	73 (83.9)		8 (80)	32 (84.2)	
Radiomics model score	0.773 ± 1.934	−2.244 ± 2.43	0.001^*^	0.349 ± 1.587		−2.629 ± 1.912	0.001^*^

*ER, estrogen receptor; PR, progesterone receptor; HER2, human epidermal growth factor receptor 2; Ki-67, antigen Ki67*.

* *p < 0.05, with significant differences for clinical characteristics of pCR group and Non-pCR group*.

### Development of the Radiomics Signature and Assessment of Its Accuracy

**Figure 2:**
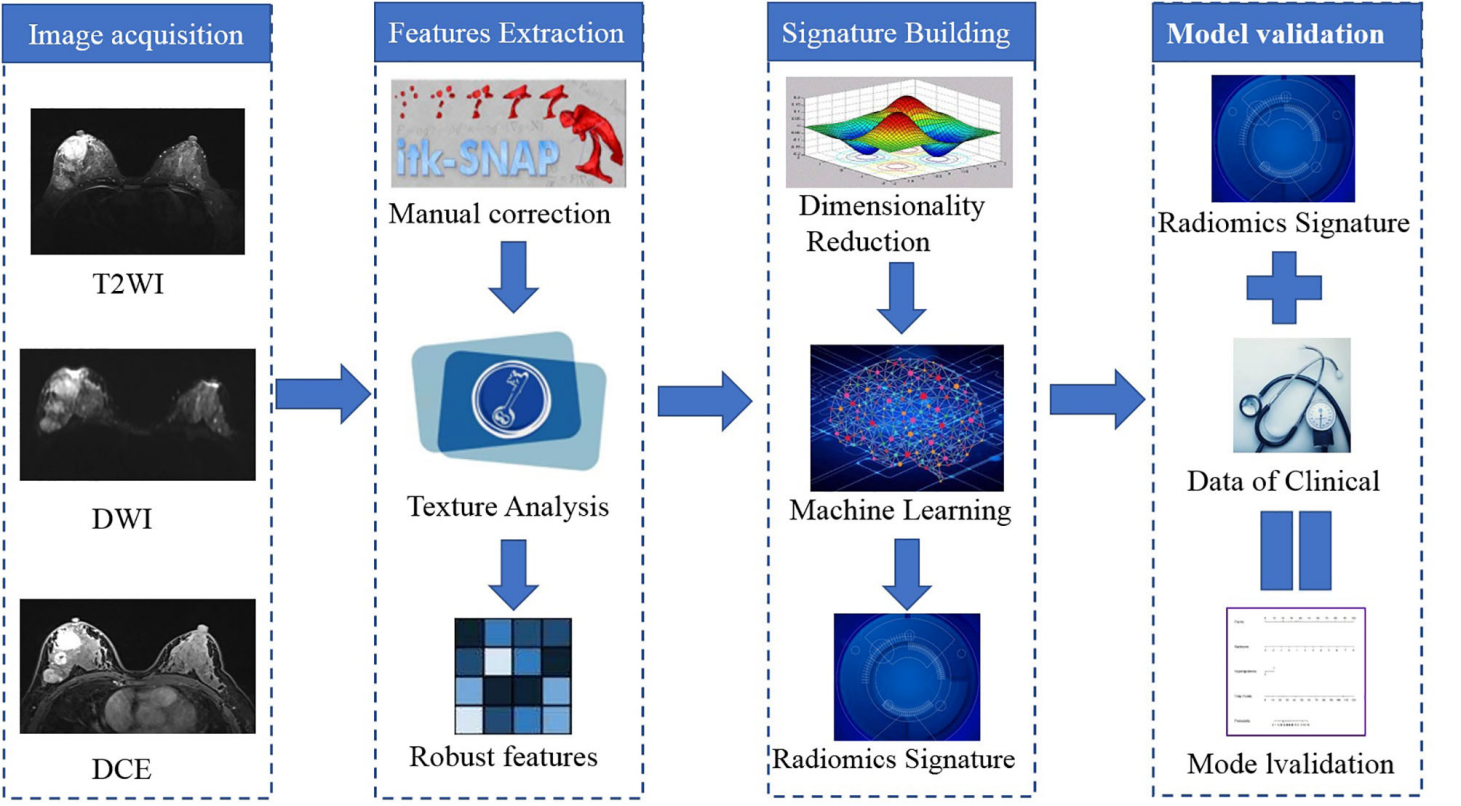
Workflow for building the radiomics signature and creating the model. T2WI, T2-weighted image; DWI, diffusion-weighted imaging; DCE, dynamic contrast enhancement.

**Figure 3:**
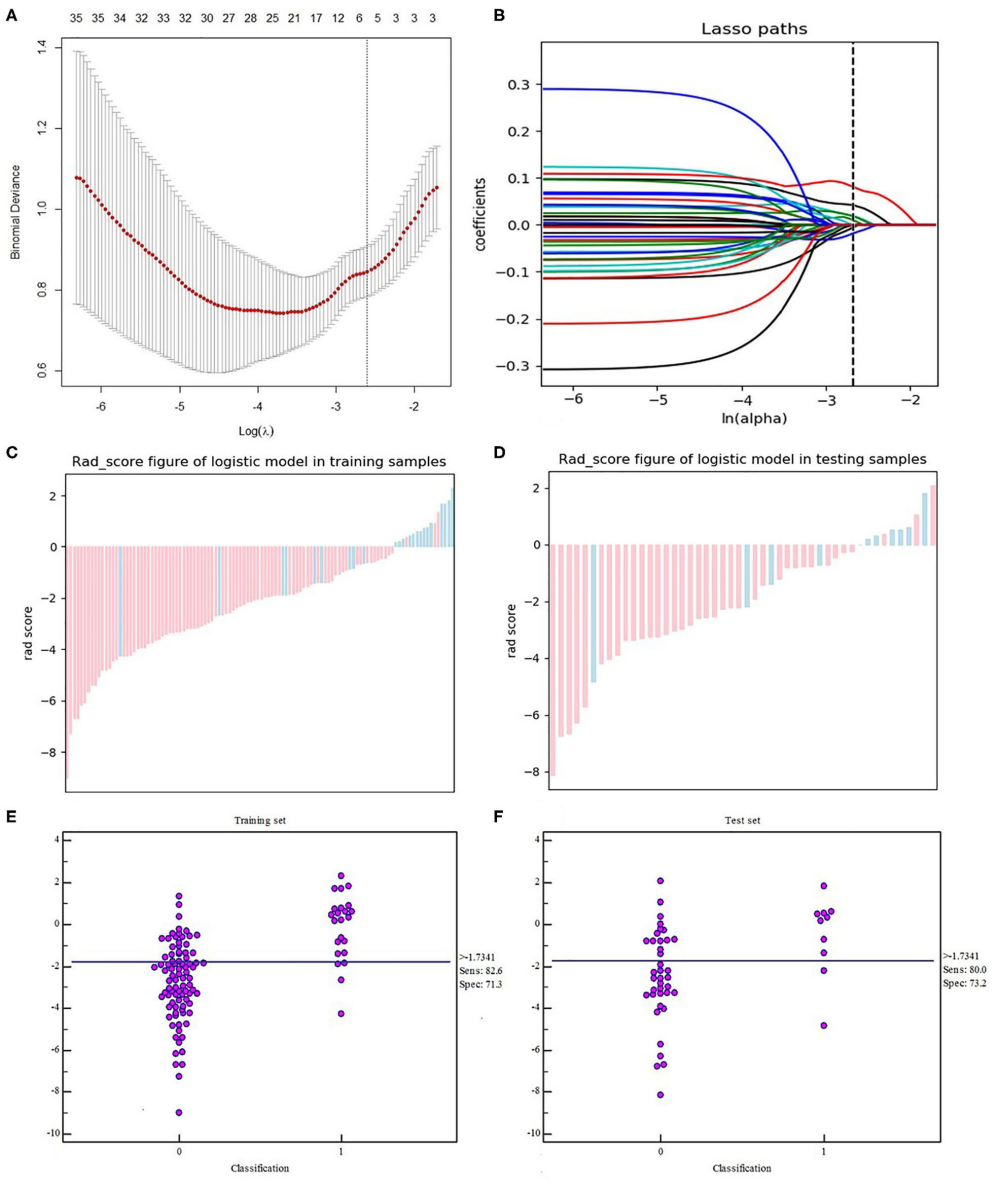
Texture feature selection using the LASSO logistic regression. **(A)** The tuning parameter (λ) selection in the LASSO model used 10-fold cross-validation via the minimum criteria. Partial likelihood deviance was plotted vs. log (λ). The dotted vertical lines were drawn at the optimal values using the minimum criteria and the 1-SE criteria. **(B)** The LASSO coefficient profiles of the 35 texture features. The vertical line was drawn at the value selected using 10-fold cross-validation in the log (λ) sequence, and six features with non-zero coefficients are indicated. Score diagrams of the radiomics signature in the **(C)** training set and **(D)** test set. Red represents non-pCR and blue represents pCR. A score >0 indicates pCR, and a score <0 indicates non-pCR. Both panels **(C,D)** show interactive dot diagrams revealing the accuracy of the radiomics signature for predicting pCR in patients of the **(E)** training set and **(F)** test set. Zero represents non-pCR, and 1 represents pCR. The horizontal line indicates the best threshold point to distinguish patients with pCR from patients with non-pCR.

**Figure 4:**
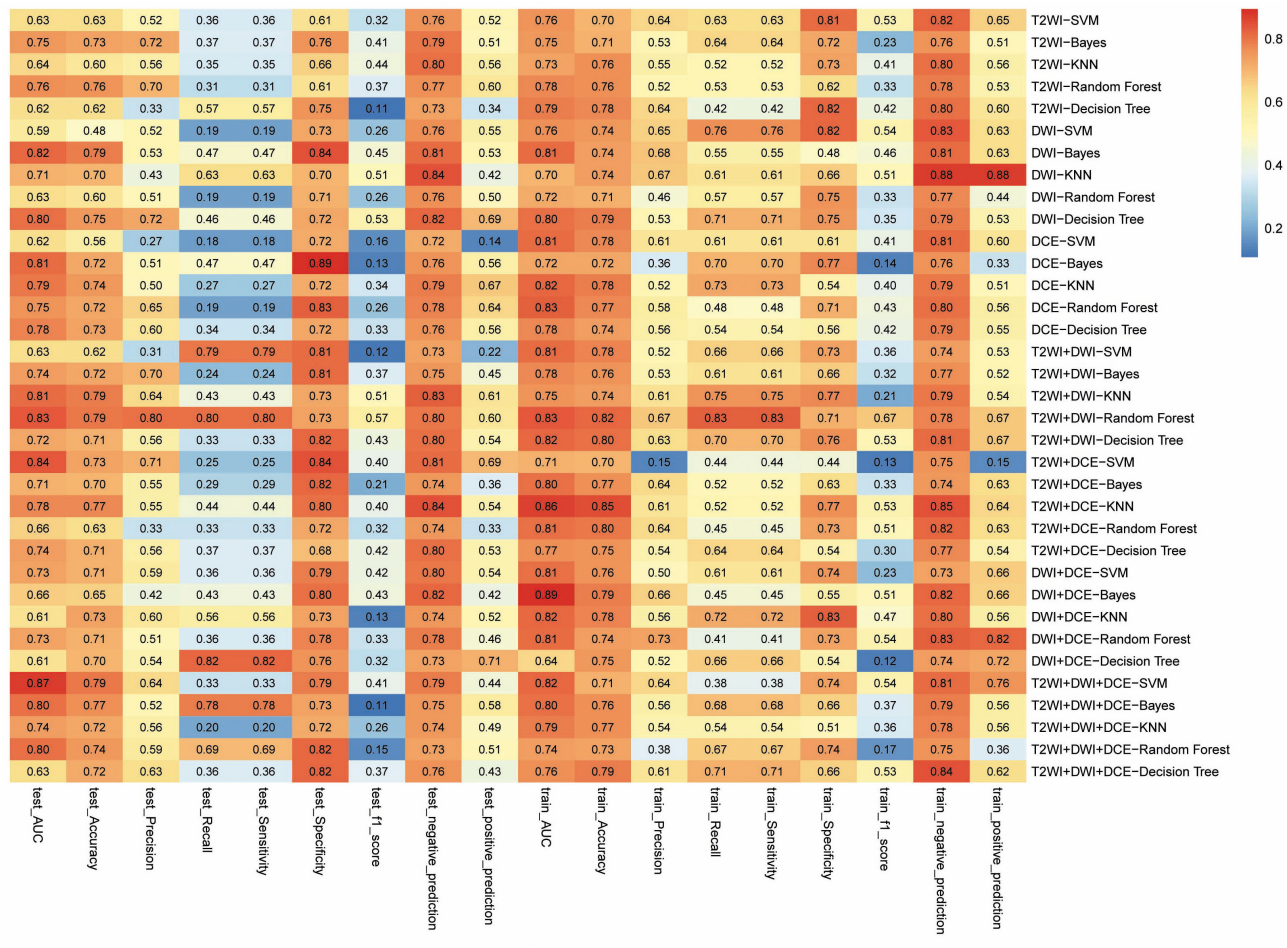
The evaluation results of radiomics signature combined features from different imaging sequences and machine learning methods. The right column of the figure shows the combination of different sequences. The horizontal coordinate shows different items to be evaluated in the training set and the test set, and the value in each frame represents the evaluated result of the items of the corresponding sequence combination. The closer the color is to red, the greater the value. In this study, the larger the AUC value represents the better combined model built by machine learning.

The radiomics workflow is shown in Figure 2. A total of 328 radiomics features were obtained from the T2WI, DWI, and DCE sequence images. Accordingly, 984 radiomics features were extracted from each patient. The optimal combination of texture features from T2WI and DWI were chosen, and the random forest method was used to construct the radiomics signature. First, 396 features were obtained from the combination of radiomic features through the detection of robustness and reproducibility. Next, 35 features with the highest mRMR rankings were selected to establish the optimal subset, and LASSO was used to reduce the dimensions of the optimal subset to obtain a total of six features, among which three of the features were from T2WI images, and three were from DWI images. Finally, the six features were used to construct the radiomics signature model. In this study, the diagnostic accuracy of the radiomic signatures constructed by the random forest showed good prediction performance in both the training set and the test set, while the difference between the two was the smallest. The radiomic signature **s**howed favorable predictive efficacy in the two sets with AUC values of 0.835 and 0.834, specificity of 71.32 and 73.21%, and sensitivity of 82.61 and 80%, respectively. Rad-scores, which were calculated using the radiomics signature formula, were significantly different between the pCR and non-pCR in training and test sets, indicating that radiomics signature has a good correlation with pCR clinical outcome, as shown in Figure 3. The evaluation results of other sequence feature combinations and different machine learning techniques used to construct the radiomics signature are shown in Figure 4. Information about the dimensionality reduction process and LASSO are shown in the Supporting Information.

### Development and Performance of the Radiomics Nomogram

**Figure 5:**
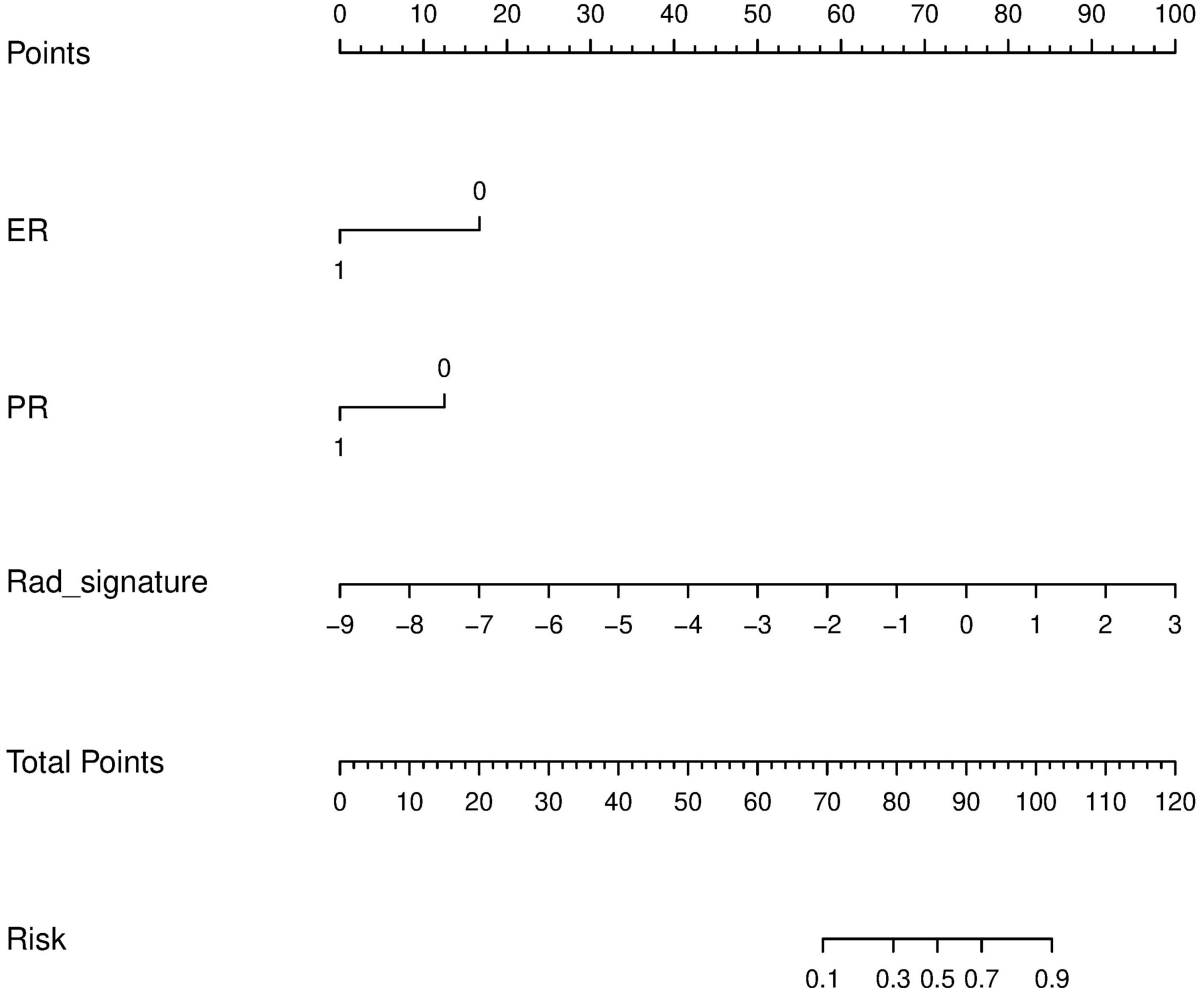
Radiomics nomogram to predict the patient with pCR. The radiomics nomogram was developed in the training set, with the rad-score, ER status, and PR status.

Univariate logistic regression analyses revealed that ER status, PR status, and the radiomics signature were independent predictors of pCR. Based on the independent predictors, multiple logistic regression was utilized to construct prediction models and the nomogram, as shown in Table 3 and Figure 5. The VIFs of ER status, PR status, and the radiomics signature were 1.017, 1.011, and 1.02, respectively.

**Table 3:** Logistic regression analysis for predicting pCR in breast cancer (BCa) patients.

**Variable**	**Univariate logistic regression**		**Multivariate logistic regression**	
	**OR (95% CI)**	***P*-value**	**OR (95% CI)**	***P*-value**
Age (per one increase)	0.955 (0.884–1.031)	0.238	NA	NA
Menopausal (No vs. Yes)	0.621 (0.145–2.656)	0.52	NA	NA
Histologic type (NST invasive carcinoma vs. Other)	2.345 (0.508–9.811)	0.118	NA	NA
ER status (Negative vs. Positive)	0.255 (0.068–0.958)	0.043^*^	0.215 (0.062–0.745)	0.015^*^
PR status (Negative vs. Positive)	0.174 (0.045–0.675)	0.011^*^	0.214 (0.064–0.715)	0.012^*^
HER2 status (Negative vs. Positive)	0.736 (0.195–2.783)	0.651	NA	NA
Clinical stage (I vs. II)	0.758 (0.167–3.446)	0.72	NA	NA
Clinical stage (I vs. III)	0.838 (0.295–2.375)	0.739	NA	NA
Ki-67 (Low vs. High)	0.913 (0.149–5.577)	0.921	NA	NA
Radiomics score (per 0.1 increase)	2.575 (1.591–4.168)	0.001^*^	2.408 (1.56–3.715)	0.001^*^

*ER, estrogen receptor; PR, progesterone receptor; HER2, human epidermal growth factor receptor 2; Ki-67, antigen Ki67*.

* *p < 0.05*.

The calibration curves showed excellent consistency between the predicted and actual pCR probabilities in the radiomics nomogram of both patient sets. The accuracy, specificity, and sensitivity of the nomogram for predicting pCR were 0.888, 79.31, and 86.96% in the training set and 0.879, 82.19, and 83.57% in the test set. The DeLong test showed AUCs of ER and PR were significantly different from that of nomogram in the training and test sets, as shown in Table 4. Therefore, the nomogram was found to perform well in both sets. Next, the Hosmer-Lemeshow test found no statistical differences between the training and test sets (*p* > 0.05), verifying the superior diagnostic accuracy of the nomogram. The pCR probability scores were estimated using the nomogram, and patients were classified into the high and low probability groups according to the Yonden index (cut-off: 0.3371), which was based on the nomogram constructed by the training set. The number of pCR cases was significantly different between the high and low probability groups (*p* < 0.0001). In addition, DCA curves showed excelled net benefits of patients in training and test sets, as shown in Figure 6.

**Table 4:** AUCs of Nomogram, radiomics Signature, ER status, and PR status for pCR prediction in Trarining and Test sets.

	**Nomogram(95% CI)**	**Signature (95% CI)**	**ER status(95% CI)**	**PR status(95% CI)**
Training set	0.888 (0.814–0.94)	0.835 (0.753–0.899)	0.658 (0.562–0.746)	0.693 (0.598–0.777)
Nomogram vs. other metrics		0.0956	<0.0001^*^	0.0015^*^
Signature vs. other metrics			0.0133^#^	0.075
ER status vs. PR status				0.06392
Test set	0.879 (0.752–0.955)	0.834 (0.699–0.926)	0.716 (0.567–0.837)	0.642 (0.491–0.775)
Nomogram vs. other metrics		0.1446	0.0384^*^	0.0066^*^
Signature vs. other metrics			0.2239	0.0366^#^
ER status vs. PR status				0.5265

*p-value refers to Delong test for the differences of AUCs between different metrics in different cohorts*,

* *p < 0.05, with significant differences for AUCs of Signature, ER status, and PR status compared with that of nomogram*.

# *p < 0.05, with significant differences for AUCs of ER status and PR status compared with that of Signature*.

**Figure 6:**
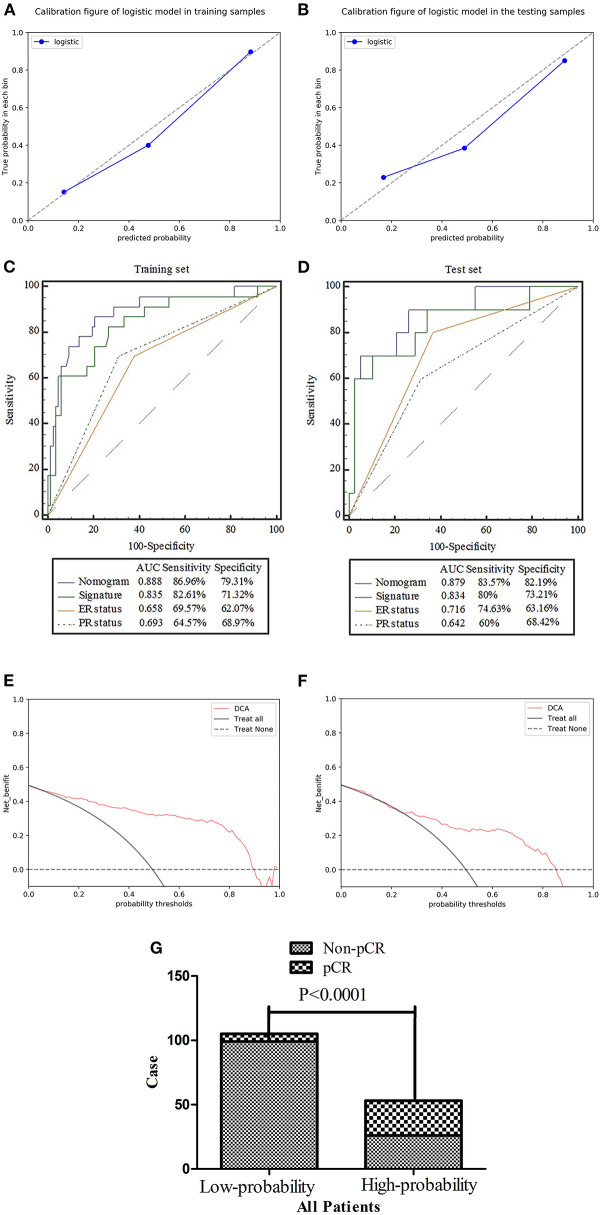
**(A,B)** Calibration of the radiomics nomogram for predicting pCR in the training and test sets. Dashed line is the reference line where an ideal nomogram would lie, while the solid line corrects for any bias in the hybrid nomogram. **(C,D)** Evaluation of the accuracy of the ER status, PR status, radiomics signature, and nomogram for predicting pCR in the training and test sets. **(E,F)** Decision curve analysis was used to show the clinical effect of the nomogram in the prediction of pCR in breast cancer (BCa) patients in training and test sets. **(G)** Probability of pCR in the high-probability group was significantly higher than that in the low-probability group (*p* < 0.0001).

## Discussion

In this retrospective study, we have quantified the prognostic abilities of different machine-learning techniques for predicting the pCR status of patients with BCa. Considering the stability and prognostic performance together, two feature selection sequences (i.e., T2WI and DWI) and the random forest classification method should be preferred for the prediction of NACT response, as they display higher prognostic abilities and stability when compared with the other models. In addition, the nomogram constructed by the radiomics signature and pathological indexes could effectively predict the patients with pCR. In total, these findings indicate that imaging-based heterogeneity using machine learning and “big data” can provide complementary prognostic information about existing risk predictors.

In recent years, several research groups have searched for clinical or molecular markers that can predict the effect of NACT for the screening of patients who can benefit from the treatment (25). However, up to now, no factor has been found that can accurately predict the efficacy of NACT. The main reason is that the accuracy of single factor prediction is limited, yet a multifactor prognostic model may overcome this limitation for BCa. Based on this principle, a multifactor pCR prediction model was developed in this study. At the same time, we also extracted radiomic features from multiparameter images, including T2WI, DWI, and DCE. In the past, the features were extracted from multiparameter images to predict the prognosis of patients by merely distinguishing features of a single and joint sequence (26, 27). However, in this study, we created a pairwise sorting combination of three sequences (T2WI, DWI, and DCE), which further expanded the scope of this type of research. In addition, our result is consistent with Yoon's results that the features most associated with the clinical outcome of pCR mainly include Inverse difference moment and short-run emphasis features (28), which indicated these two types of feature had significant difference between non-pCR patients and pCR patients with breast cancer experiencing NACT. Although the remaining features also represent the degree of heterogeneity, the exact calculation method of each feature value varies depending on the parameters. Therefore, it is difficult to explain the subtle differences of various heterogeneous parameters caused by the mathematical equations. Besides, the unique biological mechanism that may cause heterogeneity parameters still remains unclear, which may require further research to figure out its mechanism.

The combination of features from T2WI and DWI sequences showed excellent performance of diagnosis for structure radiomics signature, suggesting that a conventional sequence can be used to predict the status of pCR without requiring enhanced imaging techniques in the future, such as DCE. Although recent studies have shown that DCE-MRI is the best sequence to predict NACT response so far (29, 30). Unfortunately, in this study, we found that the prediction efficacy of the radiomics signature was not optimal after combining the features extracted from DCE sequences with those of T2WI or DWI. This is mainly due to the instability of the model performance. Although the performance of the DCE-T2WI combination or the DCE- DWI combination model in the training group is higher than that of the T2WI-DWI combination model, the performance of the model with DCE features in the test set is significantly lower to that of the training group, which indicates that the model stability is not favorable. It may be possible that we only analyzed one 2D slice in a tumor other than a whole 3D tumor in this study, which may cause the instability of the model, we will conduct a further study using 3D tumor to validate the model stability in future research.

This may be due to the different amounts of contrast agent, which can affect the selection of features. Since patients receive different amounts of contrast agent, the enhancement effects of images can affect the permeability of tissue microvascular (31). This reflects the distribution of pixels and further affects the stability of the overall construction model. DWI was previously found to be useful as a tool to measure the efficacy of NACT in patients with BCa (32). However, the diffusion characteristics of water in tissue reflect the heterogeneity of tumors, rather than some external factors, such as contrast agents, which reflect the heterogeneity of tumor by imaging features. Accordingly, it should be demonstrated that T2WI and DWI sequences provide a suitable combination for feature extraction.

The development of statistical models that use the tumor and treatment data from a single patient are better prognostic indicators than human experts (33). Hence, the radiomics-based machine-learning models may be a viable tool for clinical decision support. In this study, we use different machine learning methods to construct a radiomics signature for the extended sequence combination and obtained the highest AUC of 0.835. In another study, Braman et al. (34) also used radiomics to predict pCR status based on different machine learning methods, yet their accuracy was significantly lower than the results of this study with an AUC of 0.74. This may be due to our radiomic features having higher dimensions in our study, which can better reflect the heterogeneity of tumors (35, 36). On the other hand, this study uses a variety of potential factors combined with the radiomics signature to build the prediction model, which also improved the accuracy of this model. In this study, we found that ER and PR expression were independent predictors of patients with pCR, which is similar to other studies (37, 38). The diagnostic efficacy of the nomogram was higher than the signature after adding pathological indicators, which further demonstrates the important role of hormone receptors in the prediction of pCR status. The relationship between texture-based heterogeneity indices and pathologic prognostic factors in breast cancer was confirmed as well. Tumor heterogeneity measured on FDG PET was higher in LABC with poor prognostic pathologic features, such as hormone receptor negativity, nuclear grade 3, and triple negativity (39).

Several features differentiate our work in this study from other radiomic-based studies. First, only features with high repeatability were used in this study, making this approach less prone to the risk of overfitting. The traditional repeatability test is to test the consistency of all the extracted features among the observers, which may allow for some features with poor repeatability to be included in the study. For this reason, some of the features with low repeatability may not accurately reflect the degree of tumor heterogeneity. On the other hand, we also reduced the dimensions of the features from the MRI sequences, including mRMR of the emerging method and LASSO of the traditional dimension reduction method, which may also explain why the diagnostic efficiency of our model is better than other prediction models constructed from LASSO alone. The superiority of this model is also related to the optimal machine learning techniques used to build the model. Therefore, the optimization of the classifier of this model is another important aspect of the study, which differs from traditional radiomics studies. Lastly, our nomogram was created for clinical research. A nomogram is a statistical tool that may be utilized to assess the probability or risk of a specific clinical outcome. For clinical practice, nomograms can be used to provide detailed risk assessments for the patient, aiding in clinical decision-making for clinicians (40). In our study, we built a radiomics nomogram that combines multi-dimensional information, which dramatically improved the accuracy and effectiveness of our model. These technical advances have contributed to the improved reproducibility found in our current study.

We acknowledge several shortcomings of the current study. First, this is a study representing only one medical center, which may not be applicable to populations in other centers. However, this study utilized a contemporary cohort of patients with BCa, allowing for the derivation of a hybrid nomogram that may be used to assess more extensive and diverse populations in the future. Secondly, a single slice of the tumor was sampled for the radiomics analysis, and volumetric assessments were not performed. In a previous study, data from a single slice was found to be sufficient for this type of analysis (41). Finally, our study population was relatively small. As this is a first proof-of-principle study, future studies should employ larger patient populations.

The potential prognostic abilities of radiomics models have been highlighted in other studies. However, with the expanded use of radiomics and feature dimensions, along with machine learning techniques, higher prognostic performance could be achieved in patients with BCa. This is also reflected in our nomogram, which showed high accuracy in the prediction of patients with pCR. As a non-invasive prediction tool, it can broaden the scope in the application of genomics for cancer treatment.

## Data Availability Statement

All datasets generated for this study are included in the article/Supplementary Material.

## Ethics Statement

The studies involving human participants were reviewed and approved by Ethics committee of Zhejiang Cancer Hospital. Written informed consent for participation was not required for this study in accordance with the national legislation and the institutional requirements.

## Author Contributions

All authors listed have made a substantial, direct and intellectual contribution to the work, and approved it for publication.

## Conflict of Interest

The authors declare that the research was conducted in the absence of any commercial or financial relationships that could be construed as a potential conflict of interest.
