# Supplementary material for: Machine Learning-Based Radiomics Nomogram Using Magnetic Resonance Images for Prediction of Neoadjuvant Chemotherapy Efficacy in Breast Cancer Patients
Source: Front Oncol. 2020 Aug 13;10:1410. doi: 10.3389/fonc.2020.01410 (PMC7456979; doi:10.3389/fonc.2020.01410)
Supplement: Supplementary file 1 [file Data_Sheet_1.docx]

Machine learning-based radiomics nomogram using magnetic resonance images for prediction of neoadjuvant chemotherapy efficacy in breast cancer patients

**I. Supplementary Methods**

**1.1 Definition of Miller-Payne grade system**

Gread 1: No change or some alteration to individual malignant cells but no reduction in overall cellularit.

Gread 2: A minor loss of tumour cells but overall cellularity still high, and up to 30% loss.

Gread 3: Between an estimated 30% and 90% reduction in tumour cell.

Gread 4: A marked disappearance of tumour cells such that only small clusters or widely dispersed individual cells remain, and more than 90% loss of tumour cells.

Gread 5: No malignant cells identiﬁable in sections from the site of the tumour, only vascular fibroelastotic stroma remains often containing macrophages. However, ductal carcinoma in situ(DCIS) may be present.

**1.2 MRI acquisition**

All breast MRI scans were performed in a local hospital on a 3.0 T MRI scanner (MAGNETOM Verio A Tim System; Siemens Healthcare, Erlangen, Germany). The routine sequences included T2-weighted imaging (T2WI), diffusion-weighted imaging (DWI) and dynamic contrast-enhanced(DCE).

T2WI: repetition time/echo time= 5750/81 ms, field of view= 320×320 mm, matrix= 256×192, section thickness= 6 mm,Slice Gap=1mm,flip angle= 150°.

DWI: repetition time/echo time= 5000/85ms, field of view= 320×104 mm, matrix= 220×72, section thickness= 6 mm,Slice Gap=1mm,flip angle= 90°.

DCE: repetition time/echo time= 8/3.93ms, field of view= 448×448 mm, matrix= 340×340, section thickness= 0.8 mm,Slice Gap=0 mm,flip angle= 12°.

**1.3 Standardization of data**

Extracted texture features were standardized, which could remove the unit limits of the data of each feature and converted it into a dimensionless pure value so that the indexes of different units or orders could be compared and weighted. we used z-score normalization to make the image intensities have the properties of a standard normal distribution with $\mu\text{=0}$ and $\sigma\text{=1}$, where $\mu$ was the mean value of the images, and $\sigma$was the standard deviation. The normalized values (also called z scores) of the image intensities (*x*) were calculated as follows:

$$z=\frac{x-\mu}{\sigma}$$

After z-score normalization of image pixel intensities, the number of radiomics features arrived at 328 by AK software of GE company. Radiomics features including Histogram, Formfactor, Gray-Level Co-occurrence Matrix (GLCM) and Run length matrix (RLM). The details were described in the table below.

- 1. **Details of maximum relevance minimum redundancy (mRMR) algorithm**

The mRMR algorithm was used to select the features. First, we test the robustness and reproducibility of texture features from image of T2WI and DWI, and retain 396 features.Secondly, we selected 86 features which had the greatest correlation with the outcome of Pathological complete remission (pCR) from these robustness feature, and the correlation was analyzed between these features. Finally, 35 features with the least redundancy were selected. Detailed features can be found in Figure S1 and S2.


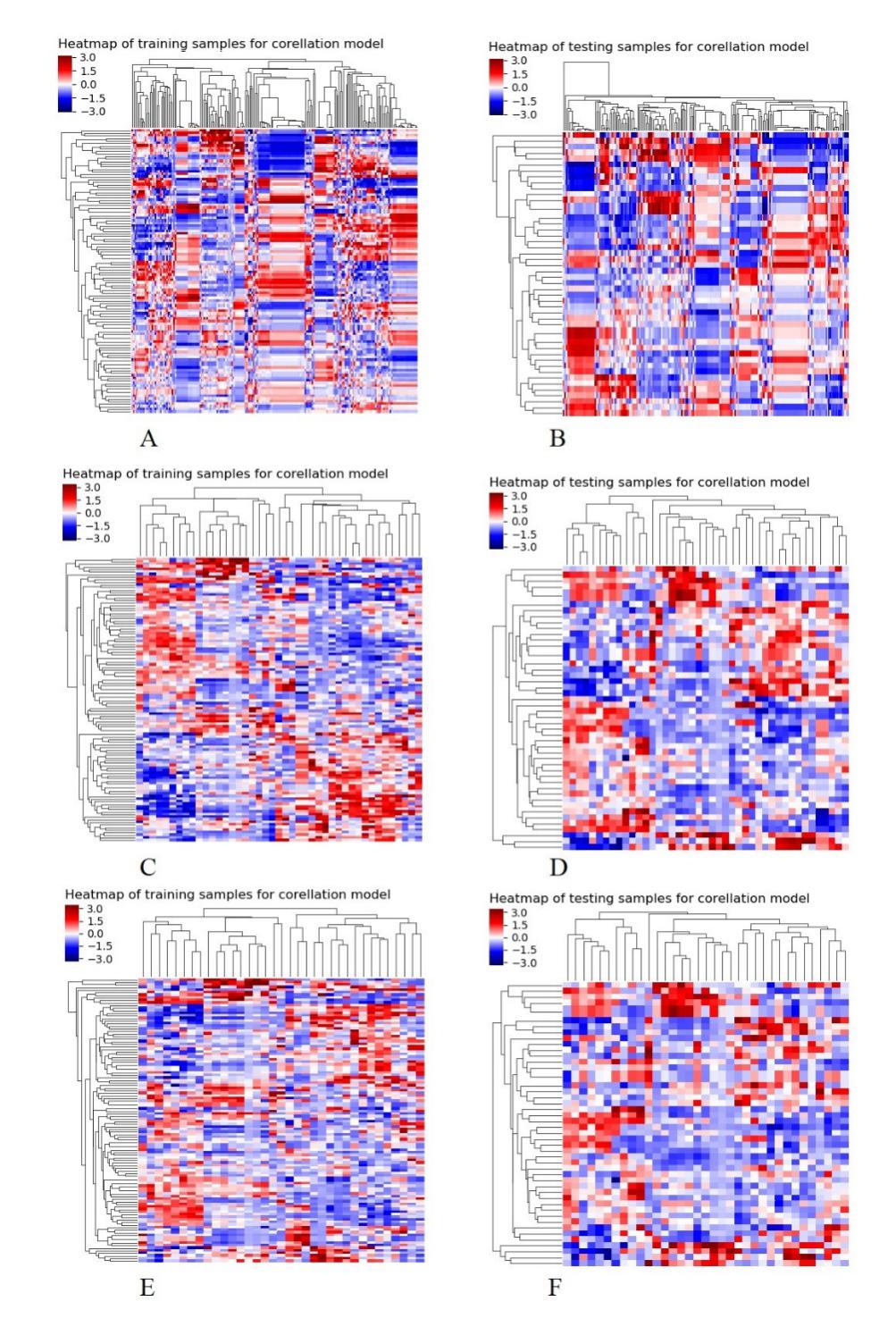


**Figure S1.** Process of mRMR for dimension reduction, A and B figures show the feature heatmap of robust features were selected from images of T2WI and DWI sequence in training and test sets, respectively. C and D figures show the feature heatmap of maximum correlation analysis between features and pCR in training and test sets. E and F figures show the minimum redundancy of feature heatmap between features and features in training and test sets, respectively.


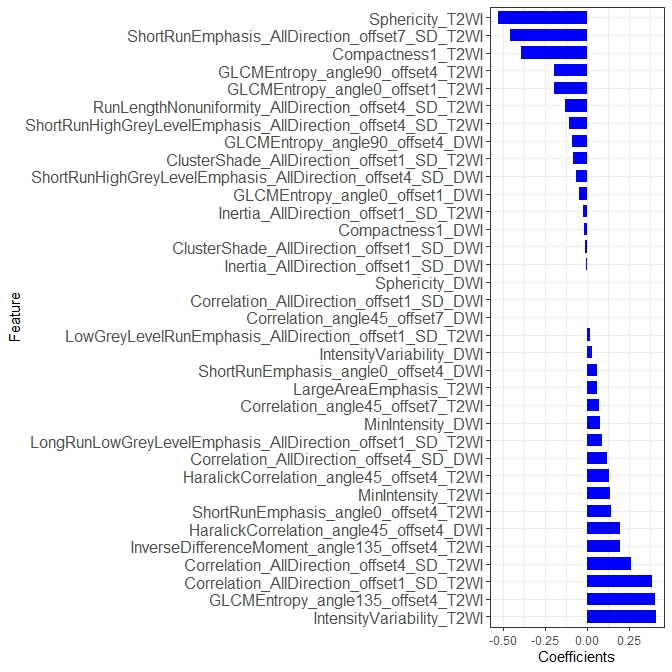


**Figure S2.** Ranking of correlation between radiomics features and pathological complete response.

**1.5. Details of absolute shrinkage and selection operator (LASSO) algorithm**

Dimensionality reduction of the selected features by LASSO. LASSO is a powerful algorithm for regression analysis with high dimensional predictors. In our study, the LASSO algorithm was combined with the different machine learning for model development. At last, We used the LASSO-Forest model to select the most important predictive features and construct a radiomics signature in the training set. Figuse S3 shows correlation between best features by LASSO. The LASSO algorithm shrinks some coefficients and reduces others to exactly 0 via the absolute constraint. Thus, LASSO is an outstanding method for feature selection by retaining the good features of both subset selection and ridge regression. In this study, LASSO selected nonzero coefficients, and six features were selected, details of the features are shown in Table S1. At last, a formula was generated using a linear combination of selected features that were weighted by their respective LASSO coefficients. The “glmnet” package in R statistical software version 3.4.1 was used for LASSO logistic regression model analysis, the specific formula is as follows.

**calculation formula:**

Rad-score = 1.29-0.174×GLCMEntropy_angle90_offset4_DWI

+0.015×Sphencity_DWI

-0.052×Compactness_DWI

-0.223×InverseDifferenceMoment_angle135_offset4_T2WI

+0.25×Correlation_ALLDirection_offer4_SD_T2WI

-0.114×ShortRunHighGreyLevelEmphasis_AllDirection_offset4_SD_T2WI

**
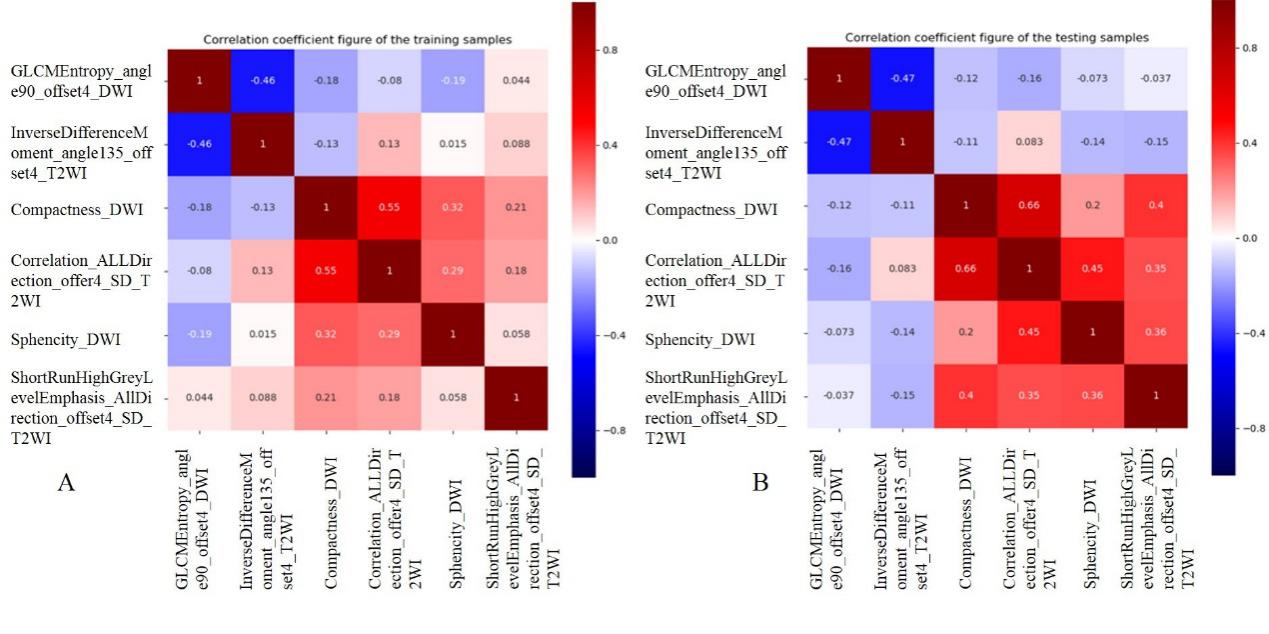
**

Figure S3. A and B figures show the correlation heatmap of between features were selected using LASSO in training and test sets, respectively.

**1.6. Decision curve analysis (DCA)**

In our study, the DCA algorithm assessed methods of the efficiency of nomogram by calculating the range of threshold probabilities in which a prediction or prognostic model was clinically useful.

DCA is a compositive method for evaluating . The theory of DCA can be illustrated by the equation below:

$$\frac{a-c}{d-b}=\frac{1-P_{t}}{P_{t}}$$

where *d* – *b* represents the influence of unnecessary predicte pCR. If predicte pCR is directed by a prediction model, *d* – *b* is the harm related to a false-positive result compared with a true-negative result. Inversely, *a* – *c* represents the consequence of rejecting beneficial predicte pCR, in other words, the harm from a false-negative result compared with a true-positive result. *P_t_* represents where the expected benefit of predicte pCR is equal to the expected benefit of refraining from no prediction.

**Ⅱ. Information of radiomics features**

**2.1 Histogram Parameters**

Histogram parameters are concerned with properties of individual pixels. They describe the distribution of voxel intensities within the CT image through commonly used and basic metrics. Let 𝑋 denote the three dimensional image matrix with 𝑁 voxels and 𝑃 the first order histogram divided by 𝑁𝑙 discrete intensity levels.

**2.2 GLCM (Co-occurrence matrices) Parameters**

The Grey level co-occurrence matrix (GLCM) $\mathbf{P}\mathbf{(}\mathbf{i}\mathbf{,}\mathbf{j}\mathbf{|}\boldsymbol{\theta}\mathbf{,}\mathbf{d}\mathbf{)}$ represents the joint probability of certain sets of pixels having certain grey-level values. It calculates how many times a pixel with grey-level **i** occurs jointly with another pixel having a grey value **j**. By varying the displacement vector **d** between each pair of pixels.

The advantage of the co-occurrence matrix calculations is that the co-occurring pairs of pixels can be spatially related in various orientations with reference to distance and angular spatial relationships, as on considering the relationship between two pixels at a time. As a result, the combination of grey levels and their positions are exhibited apparently. Therefore, it is defined as “A two-dimensional histogram of gray levels for pair of pixels, which are separated by a fixed spatial relationship”. However, the matrix is sensitive to rotation. With the change of different offsets define pixel relationships by varying directions.

The rotation angle of an offset: $0^{\circ},45^{\circ},90^{\circ},135^{\circ}$ and displacement vectors (distance to the neighbor pixel: 1, 2, 3 ...), different co-occurrence distributions from the same image of reference. GLCM of an image is computed using displacement vector d defined by its radius, (distance or count to the next adjacent neighbor preferably is equal to one) and rotational angles.


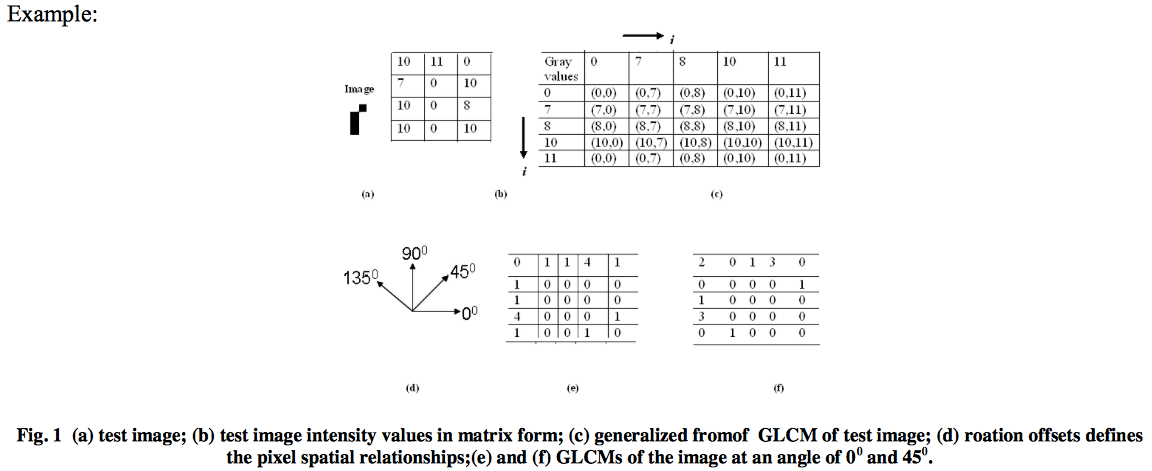


**2.3 RLM (Run-length matrices) Parameters**

### The grey level run-length matrix (RLM) $\mathbf{P}_{\mathbf{r}}\mathbf{(}\mathbf{i}\mathbf{,}\mathbf{j}\mathbf{|}\boldsymbol{\theta}\mathbf{)}$ is defined as the numbers of runs with pixels of gray level *i* and run length *j* for a given direction θ. RLMs is generated for each sample image segment having directions (0°,45°,90° &135°), then the following ten statistical features were derived: short run emphasis, long run emphasis, grey level non-uniformity, run length non-uniformity, Low Grey Level Run Emphasis, High Grey Level Run Emphasis, Short Run Low Grey Level Emphasis, Short Run High Grey Level Emphasis, Long Run Low Grey Level Emphasis and Long Run High Grey Level Emphasis.

**2.4 Form Factor Parameters**

These group of features includes descriptors of the three-dimensional size and shape of the tumor region.

**Table S1. The classification and calculation formula of texture features in three subgroups.**

| **Category** | **Feature** | **Formula** | **Describe** |
| --- | --- | --- | --- |
|  | | | |
| GLCM | InverseDifferenceMoment_AllDirection_offset7_SD | $\sum\sum\frac{1}{1+(i-j)^{2}}g(i,j)$ Where i, j are the spatial coordinates of g (i, j) | Inverse Difference Moment (IDM) is the local homogeneity. It is high when local gray level is uniform and inverse GLCM is high. IDM weight value is the inverse of the Contrast weight. |
|  | InverseDifferenceMoment_angle45_offset1 |  |  |
| RLCM | ShortRunLowGreyLevelEmphasis_AllDirection_offset4_SD | $SRE(\theta)=\frac{1}{n_{r}}\sum_{i=1}^{M} \sum_{j=1}^{N} \frac{p(i,j,\theta)}{j^{2}}$ | The grey level run-length matrix (RLM) 𝐏𝐫(𝐢, 𝐣 | 𝛉 ) is defined as the numbers of runs with pixels of gray level *i* and run length *j* for a given direction θ. RLMs is generated for each sample image segment having directions (0°,45°,90° &135°), then the following ten statistical features were derived: short run emphasis, long run emphasis, grey level non-uniformity, run length non-uniformity, Low Grey Level Run Emphasis, High Grey Level Run Emphasis, Short Run Low Grey Level Emphasis, Short Run High Grey Level Emphasis, Long Run Low Grey Level Emphasis and Long Run High Grey Level Emphasis. |
| Histogram parameter | ClusterProminence_AllDirection_offset1_SD | 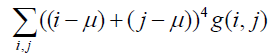 | Cluster Prominence is a measure of asymmetry of a given distribution, high values of this feature indicate that the symmetry of the image is low, in medical imaging low values of cluster prominence represent a smaller peak for the image grey level value and usually the grey level difference between the forms is small. |
| Form Factor | Sphericity Disproportion | 𝑠𝑝ℎ𝑒𝑟𝑖𝑐𝑎𝑙 𝑑𝑖𝑠𝑝𝑟𝑜𝑝𝑜𝑟𝑡𝑖𝑜𝑛=𝐴/4𝜋𝑅2 | Where 𝑅 is the radius of a sphere with the same volume as the tumor.  Where 𝑁 is the total number of triangles covering the surface and 𝑎, 𝑏 and 𝑐 are edge vectors of the triangles |
|  | Maximum3DDiameter | $A=\sum_{i=1}^{N} \frac{1}{2}\left\vert a_{i}b_{i}\times a_{i}c_{i}\right\vert$ | The maximum three-dimensional tumor diameter is measured as the largest pairwise Euclidean distance, between voxels on the surface of the tumor volume. |
